# The Ultrastructural Analysis of Human Colorectal Cancer Stem Cell-Derived Spheroids and Their Mouse Xenograft Shows That the Same Cells Types Have Different Ratios

**DOI:** 10.3390/biology10090929

**Published:** 2021-09-17

**Authors:** Michela Relucenti, Federica Francescangeli, Maria Laura De Angelis, Vito D’Andrea, Selenia Miglietta, Emanuela Pilozzi, Xiaobo Li, Alessandra Boe, Rui Chen, Ann Zeuner, Giuseppe Familiari

**Affiliations:** 1 Department of Anatomy, Section of Human Anatomy, Sapienza University of Rome, 00161 Rome, Italy; selenia.miglietta@uniroma1.it (S.M.); giuseppe.familiari@uniroma1.it (G.F.); 2 Department of Oncology and Molecular Medicine, Istituto Superiore di Sanità, 00161 Rome, Italy; federica.francescangeli@iss.it (F.F.); marialaura.deangelis@iss.it (M.L.D.A.); ann.zeuner@iss.it (A.Z.); 3 Department of Surgical Sciences, Sapienza University of Rome, 00161 Rome, Italy; vitodandrea@uniroma1.it; 4 Department of Experimental Medicine, Sant’Andrea Hospital, Sapienza University of Rome, 00185 Rome, Italy; emanuela.pilozzi@uniroma1.it; 5 Department of Environmental Medicine and Toxicology, Southeast University, Nanjing 210096, China; 101011116@seu.edu.cn (X.L.); ruichen@ccmu.edu.cn (R.C.); 6 Core Facilities, Istituto Superiore di Sanità, 00161 Rome, Italy; alessandra.boe@iss.it

**Keywords:** spheroid, xenotransplant, cancer stem cell, colorectal cancer, electron microscopy, flow cytometry

## Abstract

**Simple Summary:**

A comparative ultrastructural and flow cytometric analysis of colorectal cancer-derived spheroids and their mouse xenografts showed that they both contain the same cell types but with different ratios, reflecting the interaction of cancer cells, respectively, with the in vitro and in vivo microenvironment.

**Abstract:**

Spheroids from primary colorectal cancer cells and their mice xenografts have emerged as useful preclinical models for cancer research as they replicate tumor features more faithfully as compared to cell lines. While 3D models provide a reliable system for drug discovery and testing, their structural complexity represents a challenge and their structure-function relationships are only partly understood. Here, we present a comparative ultrastructural and flow citometric analysis of patient colorectal cancer-derived spheroids and their mice xenografts. Ultrastructural observations highlighted that multicellular spheroids and their xenografts contain the same cancer cell types but with different ratios, specifically multicellular spheroids were enriched in cells with a stem-like phenotype, while xenografts had an increased amount of lipid droplets-containing cells. The flow cytometric analysis for stem cell marker and activity showed enrichment of stem-like cells presence and activity in spheroids while xenografts had the inverse response. Our results evidence the effects on cancer cells of different in vitro and in vivo microenvironments. Those differences have to be paid into account in designing innovative experimental models for personalized drug testing.

## 1. Introduction

International Agency for Research on Cancer estimated in 2020 colorectal cancer as the third worldwide most common malignancy and second cancer-causing patient death [1]. Colorectal cancer has several sub-types and every single cancer, from a single patient, contains a heterogeneous cell population, which responded in a very different way to the conventional therapies (chemotherapy and or radiotherapy). Taking into account the patient’s characteristics, designing an effective patient-tailored treatment, is the goal of personalized medicine [2]. This approach is useful to set up preclinical studies during which drug effectiveness and safety are assessed. These kinds of studies are usually carried on established cancer cell lines monolayer cultures [3,4,5], but this model does not reproduce the incredible complexity of a whole organism, in which the cancer cell dialog with their microenvironment, with the body immune system, with other organs, even preparing a pre-metastatic niche [5,6]. In addition, after long-time culture, tumor cell lines lack the original genetic heterogeneity [7], so drug screening results may not be fully translatable with the same good outcomes on original cancer [8]. To bypass the limits of monolayer cultures different systems of in vitro 3-D culture have been developed, to better recapitulate cancer growth conditions. A recently developed 3-D culture method is the tumor-derived spheroids culture. Tumor-derived spheroids are floating spheres, derived from primary tumor cancer stem cells (CSCs), that are responsible for tumor metastasis and therapy resistance [9]. Spheroids are useful in preclinical studies on drug sensitivity and effectiveness [10]. Another experimental model that allows overcoming in vitro culture disadvantages is the patient-derived xenograft model. This procedure consists of the implantation of patient-derived cancer biopsies in the immunodeficient mouse. However, even if the mouse provides a microenvironment that lacks in vitro cultures, also this technique has some disadvantages: low rate of engraftment, it is money and time expensive, genetic and epigenetic changes due to the host are different, and being the mouse immunocompromised, interactions with the immune system are incomplete [11,12,13,14,15,16]. Both spheroids and xenotransplants are well studied by genetic and proteomic approaches [10,17], but scarce studies instead analyze their ultrastructure utilizing scanning electron microscopy (SEM) and transmission electron microscopy (TEM); in addition, the microstructure of spheroids is described in these studies only briefly [17,18,19,20]. Moreover, in the literature, no studies reported an ultrastructural investigation of patient colorectal cancer-derived spheroids compared with that of the tumor resulted from spheroids xenograft in mice. Our work aims to fill this gap investigating systematically by flow cytometry, SEM and TEM, the ultrastructural features of spheroids derived from patient colorectal cancer with that of the tumor-derived from spheroids xenograft in mice. We aim to assess, from a morphological point of view, how they are similar and how they differ in cell populations, comparing in vitro vs in vivo growth conditions. This paper presents results on the cell types characterization and distribution, and their different ratio when comparing in vitro vs in vivo environmental growth conditions.

## 2. Materials and Methods

### 2.1. CSC Isolation and Culture

Fresh human colorectal cancer tissues were obtained by the standards of the ethics committee on human experimentation of the Istituto Superiore di Sanità (authorization no. CE5ISS 09/282). Tissue samples were collected from a 63 aged year male, who underwent colorectal surgery for cancer removal. 

As previously described [10], samples were recovered immediately after the patient’s surgery, and were treated according to the following protocol: washing 2–3 times in cold saline, and transfer in Dulbecco’s modified Eagle’s medium (DMEM; Thermo Fisher Scientific, Carlsbad, CA, USA, https://www.thermofisher.com accessed on 9 September 2021). containing 3% penicillin-streptomycin-amphotericin B solution (Lonza Group, Walkersville, MD, USA, http://www.lonza.com accessed on 9 September 2021). Samples storage was at 4 °C in this medium within 24–48 h. The tissue dissociation procedure was performed as follows: cancer biopsies were washed 3–4 times in phosphate-buffered saline (PBS), they were sectioned in small fragments (0.5 mm × 0.5 mm) that were further washed twice. Samples were centrifuged at 150 g for 3 min, incubated in DMEM (Thermo Fisher Scientific) with 1.5 mg/mL collagenase type II (Thermo Fisher Scientific) and 20 μg/mL DNAse (Roche Diagnostics, Indianapolis, IN, USA, https://usdiagnostics.roche.com accessed on 9 September 2021) for 1 h at 37 °C, under shaking. At the end of the procedure, a cell suspension was obtained. The cell suspension was then filtered through a 100-μm nylon mesh and washed by two further centrifugation steps in DMEM. Finally, pellets containing cells, cell clusters and tissue fragments were resuspended in CSC medium [21] supplemented with 10 mM nicotinamide, 1 µM Y-27632 (both from Sigma-Aldrich, St. Louis, MO, USA, http://www.sigmaaldrich.com accessed on 9 September 2021), 20 ng/mL human EGF and 10 ng/mL human basic fibroblast growth factor (both from PeproTech, London, UK, https://www.peprotech.com accessed on 9 September 2021). The resulting suspension was plated in ultra-low attachment tissue culture flasks (Corning Costar, Cambridge, MA, USA, https://www.corning.com accessed on 9 September 2021), and cultured in a humidified atmosphere at 37 °C, 5% CO_2_. Every 2 to 3 days, half of the culture medium was refreshed. During the first weeks of culture, cells were periodically centrifuged at 150 g for 5 min, and the pellet was delicately passed 3 to 5 times through a 200 μL Gilson pipette tip in a small volume of the medium; then the final medium volume was added and cells were replated. Clusters of proliferating cells became evident after a variable length of time, ranging from 5 to 7 days to 3 weeks. Bacterial contamination usually developed in approximately 20% of specimens within 3–4 days of culture. After 4 weeks, were discarded the cultures in which no proliferating clusters were observed. Usually, after 3–6 weeks from isolation was needed regular culture splitting (1:2). Weekly, spheroids underwent mechanical dissociation or by incubation for 3–5 min at 37 °C with TrypLE Express (Thermo Fisher). Cultures were stored in frozen stocks, around the fifth passage, and used for in vitro and in vivo experiments within the 12th passage.

### 2.2. Animal Procedures

As previously reported [10], all animal procedures were performed according to the Italian national animal experimentation guidelines (D.L.116/92) upon approval of the experimental protocol by the Italian Ministry of Health’s Animal Experimentation Committee. Animals used were 4- to 6-week-old female NOD.Cg-Prkdcscid Il2rgtm1Wjl/SzJ (NSG) mice (The Jackson Laboratory, Bar Harbor, ME, USA, https://www.jax.org accessed on 9 September 2021). For CSC validation, 5 × 105 cells were injected subcutaneously in the flank of three replicate mice, in 100 μL 1:1 PBS/Matrigel (BD, Franklin Lakes, NJ, USA, http://www.bd.com accessed on 9 September 2021). In all the CSCs validated, xenografts were detectable within 3–5 weeks in at least 2 out of 3 mice. Palpable xenografts were extracted, formalin-fixed and paraffin-embedded. Hematoxylin and eosin-stained sections were evaluated by a pathologist (E.P), to compare xenograft histology with that of the human tumor of origin.

### 2.3. Scanning Electron Microscopy Protocol for Spheroids

Spheroids were fixed immediately upon recovery in a solution of 2.5% glutaraldehyde in Phosphate buffer 0.1 M, pH 7.4 at 4 °C for 48 h. Samples were then rinsed in phosphate buffer overnight. Samples were then post-fixed with osmium tetroxide OsO_4_ at 1.33% in H_2_O (Agar Scientific, Stansted, UK) for 2 h and washed with PBS for 20 min to remove osmium tetroxide residuals [22,23,24]. Dehydration in ascending acetone series was carried on (30%, 70%, 95%, 100% *v*/*v* × 3), followed by a critical point drying procedure (Emitech K850, Emitech, Corato, Italy). In this case, 206 spheroids were mounted on aluminum stub by carbon tape and finally sputter coated with platinum (using an Emitech K 550 sputter coater, Emitech, Corato, Italy set at 15 mA, for 3 min) and observed at Hitachi SU 4000 Field emission scanning electron microscope under high vacuum at 20 kV. SEM micrographs were acquired with a DISS5 Digital Image Scanning System (Point Electronic, Halle (Saale), Germany).

### 2.4. Flow Cytometry

For flow cytometry experiments, spheroids and mouse xenograft-derived cells were cut into small pieces, washed with ice-cold PBS and subsequently digested with TrypLE express for 15 min at 37 °C with vigorous pipetting every 5 min. Freshly isolated cells were stained with biotinylated anti-CD133, CD44v6 and anti-EpCAM, and specific secondary antibodies and monitored for the expression of GFP reporter on TOP-GFP highly expressive cells, 10 μg/mL 7-aminoactinomycin D was used for dead cell exclusion. Samples were analyzed with a FACSCanto flow cytometer (Becton Dickinson, Franklin Lakes, NJ, USA) equipped with a DIVA software. 

### 2.5. Lentiviral Infection

Primary colon spheroid cancer cells were stably transduced with TOP-GFP.mCherry (purchased from Addgene, Cambridge, MA, USA) using ProFection^®^ Mammalian Transfection System from Promega (Madison, WI, USA) following the manufacturer’s instructions.

### 2.6. Transmission Electron Microscopy Protocol for Spheroids and Xenograft

Spheroids and xenograft biopsies were fixed immediately upon recovery in a solution of 2.5% glutaraldehyde in phosphate buffer 0.1 M, pH 7.4 at 4 °C for 48 h. Samples were then rinsed in phosphate buffer overnight. Samples were then post-fixed in a solution of osmium tetroxide OsO_4_ 1.33% in H_2_O (Agar Scientific, Stansted, UK) for 2 h and washed with H_2_O for 20 min to remove osmium tetroxide residuals. Specimens underwent dehydration steps in ascending ethanol series (30%, 70%, 95%, 100% *v*/*v* × 3). Ethanol substitution with propylene oxide was performed (BDH Italia, Milan, Italy) in 50:50 ethanol 100% and Propylene oxide (two steps 20 min each). The embedding of samples was carried on in a mixture of 50:50 propylene oxide and epoxy resin Agar 100 (SIC, Rome, Italy) overnight at 25 °C (under chemical fume hood). Finally, samples were embedded in fresh epoxy resin Agar 100 (Agar scientific, Agar Scientific Ltd., Stansted, Essex, UK), and put in a stove at 60 °C for 48 h [25,26]. Semithin sections (1μm thick) were collected on glass slides, stained blue by methylene blue, to perform light microscopy observations by a Zeiss Axioskop-40, (Carl Zeiss, Oberkochen, Germany) equipped with Axiovision image acquisition software. Ultrathin sections for transmission electron microscopy observations were cut using an ultramicrotome (Leica EM UC6, Vienna, Austria). Ultrathin sections were collected on 100-mesh copper grids (Assing, Rome, Italy) stained with Uranyless© solution and lead citrate 3% solution (Electron Microscopy Science, 1560 Industry Road, Hatfield, PA, USA). Imaging was performed by a transmission electron microscope (Carl Zeiss EM10, Thornwood, NY, USA) set with an accelerating voltage of 60 kV. Images were acquired with a CCD digital camera (AMT CCD, Deben UK Ltd., Suffolk, UK). 

### 2.7. Evaluation of Spheroids Shape and Size Parameters on SEM Images

SEM images of 206 spheroids were analyzed [27] by SEM image analysis software Mountains Map 8.0 (Digital Surf, Besançon, France) Dips software (Digital image processing system, version 2.9, Point Electronic, Germany) to obtain data of the area, perimeter, Feret and Min Feret diameters. Shape parameters considered were circularity, roundness, aspect ratio and solidity. Data were statistically analyzed by Med Calc Statistical software (MedCalc Software 20.009 version Ltd., Acacialaan 22, 8400 Ostend, Belgium).

### 2.8. Ultrastructural Characterization of Spheroids and Xenograft Cell Population by Transmission Electron Microscopy

To analyze the finest details of spheroids’ ultrastructure 308 spheroids cells (belonging to 8 different spheroids) were evaluated. 118 electron microscopy micrographs captured at magnification from 10,900× to 113,000× were analyzed. An overall cell shape evaluation was carried on by two different investigators (M.R and G.F), according to cells’ main shape features (oval or columnar shape, regular/irregular outline, nucleus/cytoplasm ratio), the morphological parameters considered (Table 1) were recorded and later statistically analyzed.

### 2.9. Statistical Analysis

To analyze the finest details of spheroids and xenograft ultrastructure 308 xenograft cells (belonging to 8 different xenograft biopsies) and 206 spheroids were evaluated. The overall cell shape evaluation and the ultrastructural parameters considered were the same that form spheroids. For both spheroids and xenograft, data of each sample were recorded on tables, repeated measures ANOVA test was performed, the test of within-subjects effects and the pairwise comparisons were executed. Significance was defined as *p* < 0.05. Statistical analysis was performed by MedCalc statistical software (MedCalc Software 20.009 version Ltd., Acacialaan 22, 8400 Ostend, Belgium).

## 3. Results

### 3.1. Histological Examination of the Patient Colorectal Cancer

**Figure 1:**
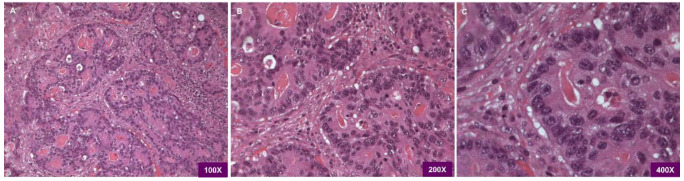
(**A**) Light microscopy H&E 100×. Histopathological examination shows the adenocarcinoma being poorly differentiated/high grade with cribriform architecture. (**B**) Light microscopy H&E 200×. the picture evidences the presence of juxtaposed gland lumens without stroma in between. (**C**) Light microscopy H&E 400×, at higher magnification loss of cell polarity is visible.

Pathological examination revealed the tumor as a large bowel poorly differentiated and high-grade adenocarcinoma, with cribriform architecture, grading pT3pN2b, G3 (Figure 1).

### 3.2. Evaluation of Patient Colorectal Cancer-Derived Spheroids Morphology by SEM

**Figure 2:**
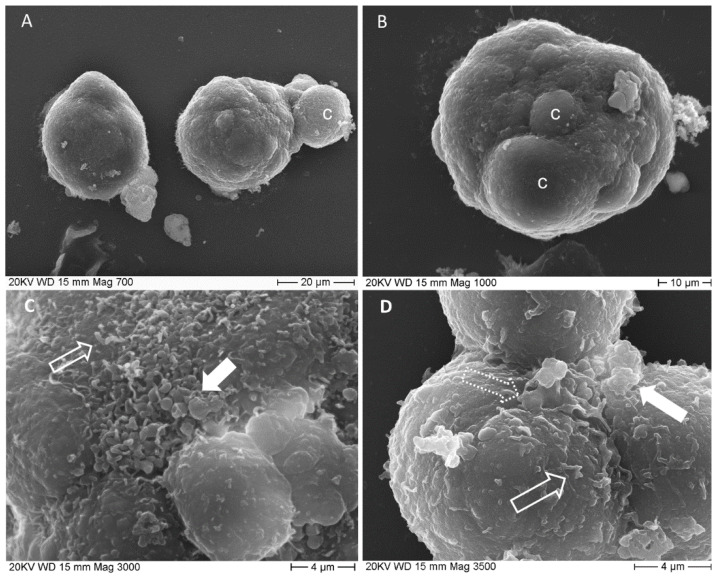
SEM micrographs of spheroids. (**A**) two spheroids are imagined at 700×, the right one has a smooth surface, the left one shows an entire cell emerging from the cell mass; c: bulging cell; (**B**) spheroid surface is irregular due to bulging cells, c, 1000×; (**C**) detailed image of spheroid surface (3000×) shows blebs, filled arrow and microvilli, empty arrow; (**D**) deep sulci delimit cells’ borders. Cells surface presents, several short processes, microvilli-like, empty arrow and pseudopodia-like, dotted arrow, together with single or multiple blebs, filled arrow.

SEM micrographs illustrate spheroids’ surface aspect (Figure 2). They appeared as cellular aggregates with a slightly irregular spheric shape (see shape descriptors and size parameters reported in Table 2 and Table 3) whose major diameter was 39.21 ± 1.51 µm and minor diameter was 32.84 ± 1.11 µm. Sometimes one of the outermost cells was observed protruding in part or almost totally from the spheroid mass (Figure 2A), as a result of the underlying mitotic activity. At the base of bulging cells sulci are evident (Figure 2B), revealing cell borders. Spheroid cell’s outer surface is smooth in some areas and rough in others, due to the presence of blebs and short microvilli (Figure 2C,D). 

### 3.3. Evaluation of Patient Colorectal Cancer-Derived Spheroids by Light Microscopy

**Figure 3:**
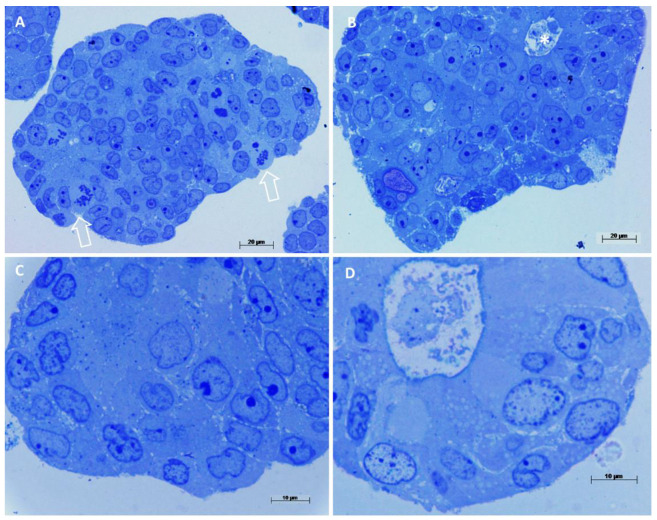
Light microscopy observations of spheroids’ semithin sections. Methylene blue staining. (**A**) Compact spheroid with several mitotic figures, arrows, cell nuclei appear dysmorphic, 400×. (**B**) In this spheroid cells are arranged circularly, forming an aberrant colonic gland, (asterisk in the lumen) 400×. (**C**) At high magnification, cells appear undifferentiated, with large nuclei, pale euchromatin and prominent nucleoli, 1000×. (**D**) Detail of an aberrant colonic gland, cells are arranged circularly around a central lumen, a brush border is visible, cells are differentiated as enterocyte-like cells, 1000×.

Light microscopy images showed smaller spheroids having a compact arrangement (Figure 3A,C), while the larger ones showed aberrant colonic glands (Figure 3B,D). The cell population is the same as the original tumor, a poorly differentiated/high-grade adenocarcinoma without goblet cells or enteroendocrine cells. Mitotic figures (*) were observed and rare apoptotic cells. Cell nuclei were almost large and oval, with pale-colored euchromatin (Figure 3D). They showed from none up to 3 nucleoli, generally 1 or 2.

### 3.4. Evaluation by Light Microscopy of the Tumor Resulted from Colorectal Cancer-Derived Spheroids Xenograft in Mouse

**Figure 4:**
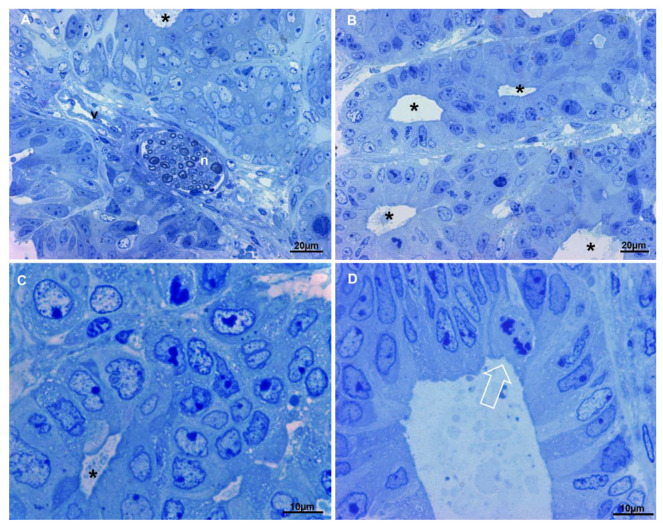
Light microscopy observations of xenograft semi-thin sections. Methylene blue staining. (**A**) In the upper part of the image cells form an aberrant gland (asterisk), in the center of the picture a nerve (n) and a blood vessel (v) are visible. Very scarce stroma is present, 400×. (**B**) Multiple aberrant glands (asterisks) are shown, they are separated by very scarce stroma, 400×. (**C**) At higher magnification, dysmorphic nuclei of cells delimiting an aberrant gland (asterisk) are evident, chromatin appears well stained and nucleoli are large. (**D**) Detail of an aberrant gland, enterocyte-like cells (on the right), less differentiated cells (on the left) and a mitotic figure (arrow) line the lumen.

The tumor resulting from cancer-derived spheroids xenograft (hereafter referred to simply as xenograft), being a tissue, obviously presents different characteristics than spheroids, which are a 3D cultured cell mass. The presence of blood vessels, nerves and fibroblasts (Figure 4A) create an extracellular environment that lacks in the spheroids culture system. In the context of the tissue, areas of necrosis and areas in hypoxia were observed, in which necrotic cells and hypoxia-suffering cells at different stages with the typical ultrastructural characteristics were present. In the non-necrotic and non-hypoxic areas, cells were arranged often in pseudocysts resembling crypts and glands of the colonic tract, with scarce stroma in between (Figure 4A–D), as in original cancer from which they derive. No goblet or enteroendocrine cells were visible, as well as in the spheroids from whom the xenograft originates and in the patient cancer, so also the xenograft reproduces the same type of cancer of the human patient. Cells are arranged in clusters or form numerous aberrant colonic glands, layered by one of two epithelial cells rows (Figure 4B–D). In those glands cells similar to enterocytes, with a brush border and a columnar shape may be observed, as well as more oval cells, with large oval and indented nucleus, which do not open into the gland lumen. Cell nuclei are dysmorphic, with large nucleoli and the chromatin appears well stained and with a more granular texture to that of spheroids cells. In particular, heterochromatin aggregates along the inner aspect of the nuclear membrane; this structure is well stained and nuclei appear to have a well-marked border.

### 3.5. Ultrastructural Characterization of Patient Colorectal Cancer-Derived Spheroids’ Cell Population by Transmission Electron Microscopy

**Figure 5:**
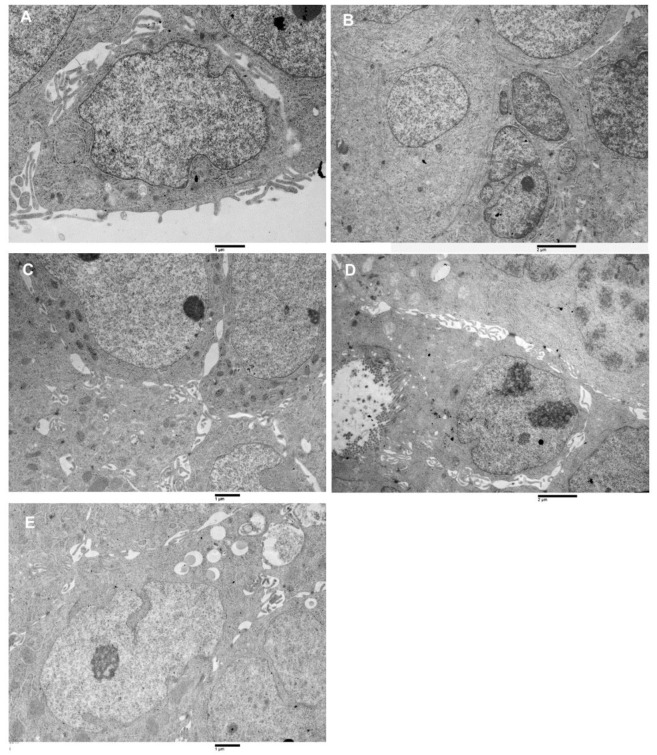
Transmission electron microscopy images of spheroids cell types. (**A**) Type-A cell, large nucleus, oval shape very finely dispersed chromatin, small cytoplasm amount, poor of organelles, 16,000×. (**B**) Cluster of type B cells, the cytoplasm is more abundant than in type A, presence of RER, 10,500×. (**C**) Group of type C cells, intercellular spaces are visible, in which cells protrusions develop. Numerous mitochondria characterize the cytoplasm. 14,000×. (**D**) A type D cell, enterocyte-like is observed. It has microvilli on the apical pole and a large nucleus (with two prominent nucleoli) in the basal part. Intercellular spaces separate this cell from its surroundings. Altered junctional complexes in the lateral domain and the absence of basal binding complexes are observed 16,000×. (**E**) Type E cell is represented. It has a large nucleus, a nucleolus, cytoplasm with RER and mitochondria, its peculiar characteristic is the presence of lipid droplets in the cytoplasm, 14,000×.

Spheroids are populated by cells, which presented common features and peculiar characteristics (Figure 5A–E). Common features include nuclear dysmorphism and ribosome abundance. The nuclear envelope showed invaginations and pockets, focal or complete absence of peripheral heterochromatin, and dispersed interchromatin; large nucleoli with reticulated architecture were observed, as well as cytoplasm filled with ribosomes. The presence of peculiar characteristics allowed classify the spheroids cell population in 5 main cell types, from the more undifferentiated (Figure 5A) towards the more differentiated cell types (Figure 5D,E):

Type A: cells showed an irregularly oval shape, they have a large nucleus (nucleus cytoplasm ratio > 1) containing generally 0 or 1 nucleolus, the chromatin was finely dispersed. The cytoplasm was generally poor of organelles (Figure 5A).

Type B: cells appeared as irregularly fusiform, the nucleus was large (nucleus cytoplasm ratio >1 ) and it generally presented 0 or 1 nucleolus. The cytoplasm was rich in rough endoplasmic reticulum (RER) (Figure 5B).

Type C: cells showed an irregularly columnar shape, their nucleus was smaller than that of type A and B cells (nucleus cytoplasm ratio ≤ 1) and contained from 0 up to 3 nucleoli. The cytoplasm contained a rich amount of RER and mitochondria (Figure 5C).

Type D: Cells had the aspect of differentiated enterocytes, even if with abnormal microvilli and altered lateral domain junctional complexes. Nucleus cytoplasm ratio was <1, nucleoli number was variable from 0 up to 3, and cytoplasm was filled with abundant RER, mitochondria and ribosomes. Being absent the basement membrane, the basal domain junctional complexes were absent (Figure 5D).

Type E. This cells type appeared as having an irregularly oval shape, with oval or irregular nucleus (nucleus cytoplasm ratio ≤ 1). The cytoplasm contained small amounts of RER and mitochondria but was rich in lipid droplets (Figure 5E).

### 3.6. Ultrastructural Characterization of Xenograft Cell Population by Transmission Electron Microscopy

**Figure 6:**
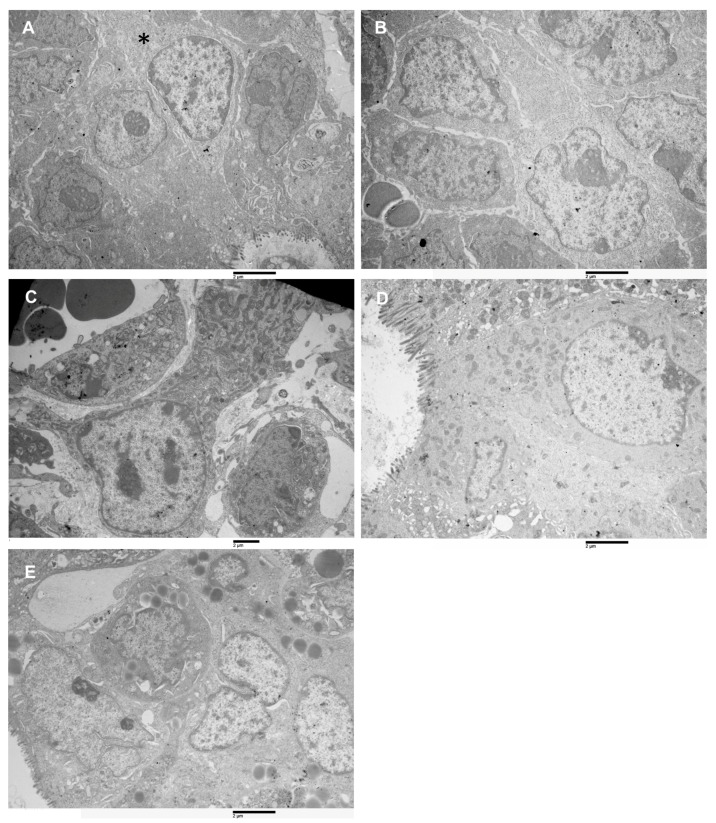
Transmission electron microscopy images of xenograft cell types. (**A**) Type-A cell (asterisk), among Type-D cells, 16,000×. (**B**) Cluster of type B cells, the cytoplasm is more abundant than in type A, presence of RER, 10,500×. (**C**) A type C cells, loosely attached to its surroundings, the cell has an irregularly columnar shape. Numerous mitochondria and abundant RER characterize the cytoplasm. 14,000×. (**D**) Type-D cells, enterocyte-like, are observed. A longitudinally sectioned cell is visible; it shows microvilli on the apical pole and numerous mitochondria in the apical cytoplasm. It has a large nucleus (with chromatin aggregates along the nuclear membrane) in the basal part. It is strictly adherent to its surroundings, but it does not lie on a basal membrane. 16,000×. (**E**) Cluster of type-E cells are represented. They have a large nucleus, a nucleolus, cytoplasm with RER and a variable amount of mitochondria, abundant and lipid-filled cytoplasmic droplets characterize this cell type, 14,000×.

Being the xenograft derived from spheroids, an ultrastructural evaluation of its cells was conducted to evaluate how xenograft cells are similar and how they differ from the spheroid’s cells (Figure 6A–E). Even in this case general features and specific characteristics were found. General characteristics include the presence of dysmorphic nuclei, with a higher degree of variation to spheroids. Even if the nuclear envelope presented invaginations and pockets, the heterochromatin has a different arrangement, peripheral heterochromatin aggregate on the inner aspect of the nuclear membrane was present and sometimes abundant, and also heterochromatin aggregate dispersed in the interchromatin were observed. Large nucleoli with reticulated architecture were present, as well as cytoplasm filled with ribosomes. Mitochondria generally showed morphological mild grade alterations (cristae reduction or swelling) due to the low oxygen content in the cancer tissue environment. Peculiar characteristics allowed recognition in the xenograft of a cell population containing the same 5 cell types identified in the spheroids with interestingly different ratios (Figure 6A–E). 

### 3.7. Data Statistical Analysis

**Figure 7:**
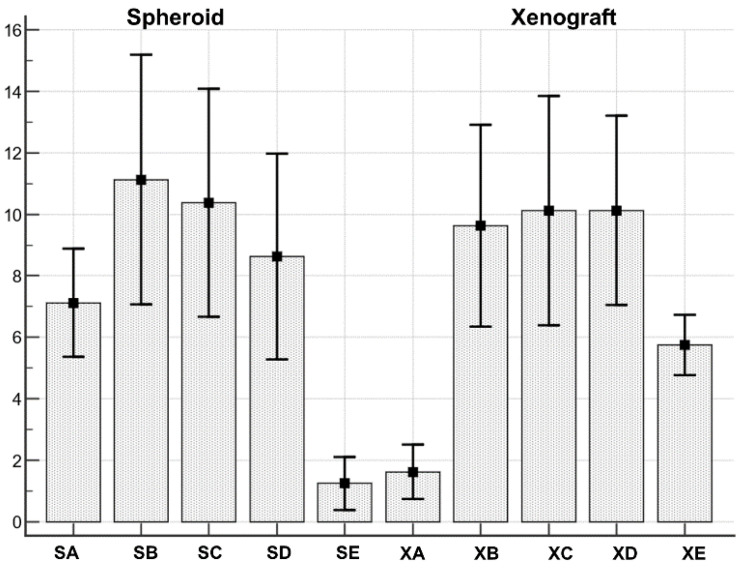
Both spheroids and xenograft cell types series were normally distributed. SA: Spheroid cell type A; SB: Spheroid cell type B; SC: Spheroid cell type C; SD: Spheroid cell type D; SE: Spheroid cell type E. XA: Xenograft cell type A; XB: Xenograft cell type B; XC: Xenograft cell type C; XD: Xenograft cell type D; XE: Xenograft cell type D.

Spheroids and xenograft cell types series were normally distributed (Figure 7, Table 4).

The Anova test demonstrated the existence of a statistically significant (F = 9.69; *p* < 0.001) difference between cell types distribution in spheroid and xenograft (Table 5).

In particular, when pairwise comparisons were performed (Table 6), cell type A resulted differently distributed in spheroid vs xenograft (more abundant in spheroids, mean 7.12 ± 0.74, than in xenograft mean 1.62 ± 0.37; significance *p* = 0.0061). In addition, cell type E resulted differently distributed (more abundant in xenograft, mean 5.75 ± 0.41 than in spheroids, mean 1.25 ± 0.36; significance *p* = 0.0211). Cell types B, C and D were not differently distributed between spheroids and xenografts. 

### 3.8. Phenotypic Analysis by Flow Cytometer of Spheroids and Xenograft Cells Reveal a Decrease in Stem Cell Number and Activity in Ex Vivo Sample

**Figure 8:**
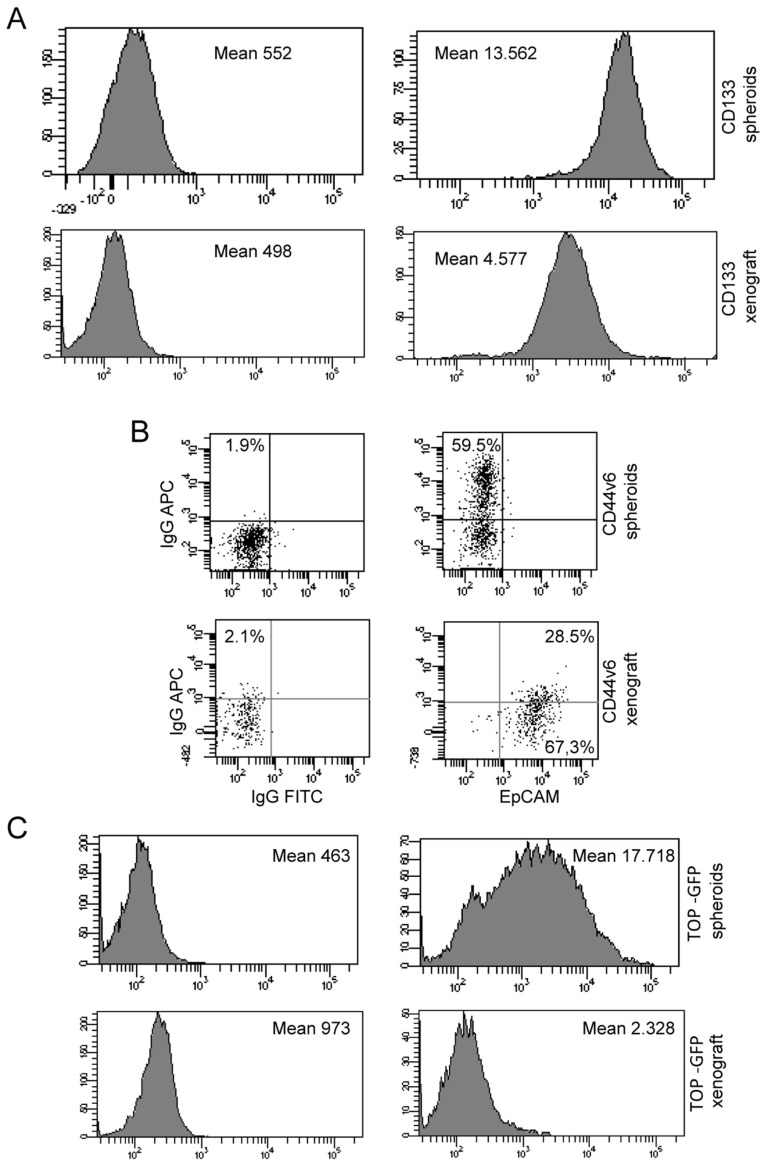
Phenotypic analysis by flow cytometry of spheroids and xenograft cells. (**A**) Flow cytometry analysis of CD133 expression of spheroids (upper panel) and CD133/EpCAM expression of xenograft (lower panel). (**B**) Flow cytometry analysis of CD44v6 expression of spheroids (upper) panel and CD44v6/EpCAM expression of xenograft (lower panel). (**C**) Flow cytometry analysis of TOP-GFP expression of spheroids (upper) panel and xenograft (lower panel).

By flow cytometric analysis we observed that in the xenograft cells sample decrease the stem cell content if compared with in vitro cultured spheroids. Figure 8A,B (lower panel) show that colorectal cancer stem cells marker CD133 and CD44v6 are decreased in xenograft cells. 

To evaluate if there was a drop-in stem cell activity in xenograft, we investigated TOP-GFP expression in vitro cultured spheroids and xenograft cells transduced with a TOP-GFP.mCherry vector. We observed that the Wnt activity is reduced in xenograft cells, (Figure 8C lower panel), as it was for CD133 and CD44v6 markers.

## 4. Discussion

In this paper, we provide a comparative analysis, by TEM, SEM and flow cytometry, of patient colorectal cancer-derived spheroids with that of cancer induced by their mouse xenograft. In literature, few studies are present reporting on spheroids ultrastructure, moreover, they lack the character of systematicity [17,18,19,20]. TEM study of [17], showed that tumor-derived spheroids cells preserve, to some extent, a polarized epithelial-like character, with a considerable variation in the shape and size of spheroids cells. In the study of [18], spheroids derived from the co-culture of colonic adenocarcinoma cells and normal colonic fibroblasts were observed by transmission electron microscopy. Colonic adenocarcinoma cells showed characteristics of intestinal cells: microvilli, main nuclei with irregular contour, and mildly coarse chromatin and nucleoli compared to colonic fibroblasts with no microvilli, and other cell types having nuclei with a smooth nuclear membrane and dispersed chromatin with the larger nucleolus. Fibroblasts surrounded cancer cells located at the periphery of the spheroid were observed, interacting with them. These and other data demonstrated that cells interaction was essential for the tumorigenicity of cancer cells as well as for tumor propagation [18]. The paper of [28] presents the results of an ultrastructural investigation of colon cancer spheroids cultured alone, compared with colon cancer spheroids cultured with fibroblast in collagen gels. Authors found that co-cultured spheroids had well-developed junctional complexes and expressed a large pattern of mucins if compared with that of spheroids cultured alone. In the paper of [19], the ultrastructure of xeno-free pre-vascularized spheroids was compared to that of spheroids cultured in a PBS-supplemented medium. Similar ultrastructural features were detected in spheroids from both conditions, with elongated cells at the more peripheral layers and accumulation of extracellular matrix components; spheroid cells exhibited typical organelles. In the paper [17] authors also observed by scanning electron microscopy on the spheroid surface that revealed, in addition, a somewhat variable spheroid outer appearance, ranging from a largely smooth surface to a blebbier aspect, even for spheroids derived from the same tumor. In the paper of [20], scanning electron microscopy observations on spheroids derived from HT-29 and Caco-2 colorectal cell lines were presented. Spheroid derived from these cell lines had an outer surface, on both cases, smooth and without any plasma membrane projections or microvilli, so that it was hard to distinguish individual cells. In addition, hollow structures were observed in Caco-2 spheroids, whereas HT-29 spheroids showed conglomerate appearance. No studies were found in literature discussing the ultrastructure of patient colorectal cancer-derived spheroids and their mouse xenograft so, our study aimed to fill this gap, using electron microscopy observations and cytofluorimetric analysis. SEM observation of the outer surface of our spheroids showed some samples having blebs and short microvilli, as observed by [17], and some others having a smooth surface, according to [20]. As assessed in [29], important morphological parameters to characterize spheroid growth and response to treatments are their size and circularity. Even if the production of a homogeneous spheroids population is still challenging, the spheroids population we analyzed resulted in having the shape descriptor circularity near to 1, the value of a perfect circle and similarly-sized elements in which diameters had a very low standard deviation. The overall homogeneity of spheroids we analyzed strengthens our cell types classification by TEM and also translates this significance to xenograft cell types classification. The innovativeness of our work consists in the comparative analysis we performed by scanning and transmission electron microscopy, together with flow cytometry of patient tumor-derived spheroids and cancer induced by their xenograft in the mouse. Our TEM and SEM analyses results showed that both experimental models reproduce the colorectal cancer of origin (poorly differentiated, high-grade adenocarcinoma with cribriform architecture) at the ultrastructural level, but highlighted some important differences in cell composition (five types of cells were detected). In multicellular spheroids, approximately 50% of the cells exhibited features typical of undifferentiated cells, such as a large nucleus and scant cytoplasm containing few organelles. Such high content in stem-like cells is consistent with the virtual absence of differentiative signals present in the microenvironment, as spheroid cultures are kept in a serum-free medium supplemented with epidermal growth factor (EGF) and basic fibroblast growth factor (bFGF). EGF and bFGF are commonly added into stem cell cultures to enhance their proliferation capacity while maintaining stem cells’ undifferentiated state.

By contrast, the tumor resulted from spheroids xenografts in mice exhibited a strong reduction in stem-like cells, consistent with the spatial arrangement into abortive colonic structures such as aberrant glands and pseudocysts. The loss of stem-like cells and the more organized architecture of xenograft may be due to the influence of a much more complex microenvironment as compared to the in vitro culture, including blood vessels, nerves, stromal cells and fibroblasts. These cellular elements, together with the heterogeneous availability of oxygen and nutrients, create a more physiological environment promoting the differentiation of tumor cells. Interestingly, xenografts contained a significantly increased amount of cells enriched in lipid droplets, which are involved in multiple cellular functions. First, lipid droplets are enriched in cells undergoing nutrient deprivation and are responsible for preventing hypoxic damage to mitochondria, for example in cells with a high autophagic flux [30]. Secondly, lipid droplets facilitate the communication between different organelles and act as vital hubs of cellular metabolism, reflecting the more dynamic microenvironment found in vivo. Third, lipid droplets are involved in the crosstalk between tumor cells and their microenvironment [31]. Finally, lipid droplets are associated with several hallmarks of cancer, being involved in rapid cell proliferation, hypoxia response and epithelial-to-mesenchymal transition [31]. As the main application of experimental colorectal cancer models is the preclinical testing of candidate therapeutic drugs, the relative differences in cell types between spheroids and tumor xenografts should be useful in model choice and interpretation of the results. Spheroids may be more appropriate for testing putative anti-cancer stem cells drugs, while xenografts allow evaluating the effect of drugs on a more physiological collection of tumor cells including lipid droplet-enriched cells. Colon cancer spheroids derived from primary human tumors have been previously demonstrated to be enriched in CSCs [32,33,34]. In addition, TOP-GFP system provides a functional evaluation of stem cell content in colorectal cancer by, recapitulating the expression of the Wnt target TCF linked to GFP [35]. Further, we observed using colon cancer stem cells marker CD133 [32] and CD44v6, a marker of constitutive and reprogrammed CSCs driving colon cancer metastasis [36] that the xenograft-derived cells have a lower stem cell content and activity. Finally, to evaluate if there was a drop in stem cell activity in xenograft-derived cells, we investigated TOP-GFP expression in vitro and in vivo in cells transduced with a TOP-GFP.mCherry vector [35]. According to results with stem cell markers CD133 and CD44v6, in the ex vivo samples, there is a lower Wnt activity. Taken altogether, our observations highlight that the ultrastructural differences in spheroids and xenografts cells, that allow classifying them in different cell populations, are in agreement with flow cytometry data on cell stem content and activity. Those differences result from the influence of in vitro culture and in vivo microenvironment conditions. The knowledge of such differences is useful to improve the design of experimental models more adherent to the in vivo condition to be used in preclinical colorectal cancer research.

## 5. Conclusions

This study contains a comparative analysis, by TEM, SEM and flow cytometry of multicellular spheroids derived from colorectal cancer surgical specimens and the corresponding tumor xenografts. We observed an enrichment of stem-like cells in spheroids while xenografts were characterized by lower stem cell content and activity and showed lipid droplet-containing cells, possibly reflecting complex interactions with the in-vivo tumor microenvironment. Our result will aid in the design of innovative experimental models for personalized drug testing.

## Figures and Tables

**Table 1:** Morphological parameters that are considered in cell type evaluation.

Cell Compartment	Morphological Parameters
Membrane	Microvilli, apical binding complex; basal and lateral domains
Cytoplasm	Types and morphology of organelles, filaments and inclusions.
Nucleus	Shape, number of nucleoli; chromatin aspect

**Table 2:** This table shows summary statistics values for spheroids shape descriptors.

Descriptors	Mean	Std. Error	95% CI
Circularity	0.94	0.006	0.92 to 0.95
Roundness	0.87	0.008	0.86 to 0.89
Aspect Ratio	1.17	0.02	1.12 to 1.22
Solidity	0.99	0.001	0.98 to 0.99

206 spheroids were measured.

**Table 3:** This table shows summary statistics values for spheroids size parameters.

Parameter	Mean	Std. Error	95% CI
Area	967.75 µm^2^	59.95 µm	844.26 to 1091.24 µm
Perimeter	112.21 µm	3.46 µm	105.07 to 119.35 µm
Feret diameter	39.21 µm	1.51 µm	36.10 to 42.32 µm
Min Feret diameter	32.84 µm	1.11 µm	30.55 to 35.14 µm

206 spheroids were measured.

**Table 4:** This table shows summary statistics values for spheroids and xenograft cell types.

Factor	Mean	Std. Error	95% CI
Spheroid cell type A	7.1250	0.7425	5.3692 to 8.8808
Spheroid cell type B	11.1250	1.7159	7.0676 to 15.1824
Spheroid cell type C	10.3750	1.5691	6.6647 to 14.0853
Spheroid cell type D	8.6250	1.4134	5.2828 to 11.9672
Spheroid cell type E	1.2500	0.3660	0.3846 to 2.1154
Xenograft cell type A	1.6250	0.3750	0.7383 to 2.5117
Xenograft cell type B	9.6250	1.3879	6.3431 to 12.9069
Xenograft cell type C	10.1250	1.5748	6.4013 to 13.8487
Xenograft cell type D	10.1250	1.3016	7.0472 to 13.2028
Xenograft cell type E	5.7500	0.4119	4.7761 to 6.7239

**Table 5:** Repeated measures ANOVA-Test of Within-Subjects Effects.

Source of Variation	Sum of Squares	DF	Mean Square	F	P
Sphericity assumed	941,550	9	104,671	9.69	<0.001
Greenhouse Geisser	941,550	3.711	253,701	9.69	<0.001
Huynh-Feldt	941,550	8.420	111,827	9.69	<0.001

The gray background underlines that the *p*-value is significant.

**Table 6:** Repeated measures ANOVA-Pairwise comparisons.

Factor		Mean Difference	Std. Error	*p*	95% CI
Spheroid cell type A vs.	Xenograft cell type A	5.500	0.732	0.0061	1.613 to 9.387
Spheroid cell type B vs.	Xenograft cell type B	1.500	2.619	1.0000	−12.405 to 15.405
Spheroid cell type C vs.	Xenograft cell type C	0.250	1.461	1.0000	−7.507 to 8.007
Spheroid cell type D vs.	Xenograft cell type D	−1.500	1.195	1.0000	−7.847 to 4.847
Spheroid cell type E vs.	Xenograft cell type E	−4.500	0.732	0.0211	−8.387 to −0.613

The gray background underlines that the *p*-value is significant.

## Data Availability

Micrographs and tables are stored in our University and are available upon request.
